# A Case of Prostatic Calculus

**Published:** 1887-11

**Authors:** J. W. Philpott

**Affiliations:** Fort Madison, Iowa


### *A CASE OF PROSTATIC CALCULUS.*

By J. W. PHILPOTT, M. D., Fort Madison, Iowa.

Prostatic calculi are of such infrequent occurrence, that the following brief report of a case may be of interest to the profession:

T. S., aged sixty-two years, of good habits, had been a great sufferer for several years with a disease referred to the bladder by physicians whom he consulted. His business compelled him to ride on horseback, until he was compelled to relinquish that kind of physical exercise on account of the pain and inconvenience following every journey. The symptoms were: irritation and inflammation of the prostatic portion of the urethra and neck of the bladder. The urine was acid in its reaction, and micturition gave him great pain. The muscular walls of the bladder were atrophied to an extent that compelled the use of a catheter to empty the organ several times daily.

The treatment consisted usually of alkalies by the stomach, and the washing out of the bladder with warm water, and the biborate of soda or a weak solution of carbolic acid. Failing to obtain relief, he visited New York, to consult a specialist. Two of the most eminent genito-urinary surgeons of the metropolis, whom he consulted, after a thorough examination of the case, diagnosed an enlarged prostate, and advised the continuance of the treatment which his attending physician had previously instituted. About ten days after his return to Iowa, quite disheartened from his failure to obtain the sought-for relief from suffering, which had at that time become well nigh unendurable, while urinating, a solid calculus passed away and fell into the vessel. This calculus was about the size of a large pea, and of an oblong form. His symptoms were ameliorated at once, the painful urination passed away, and all sensations, referrable to bladder and urethra, which had made his life a burden, vanished. His health, impaired by the terrible sufferings of years, was restored, and he became a new man physically. The calculus was examined in its chemical composition by Prof. Geo. H. Shafer, Commissioner of Pharmacy of Iowa, who reported to me that it consisted of carbonate of lime, thus proving that it was a prostatic calculus.

This case is of sufficient interest to report. The rarity of cases of this type, and the favorable termination, will explain the motives actuating the writer in reporting it.

